# Association between depression and blood pressure in community-dwelling older adults: focus on Bushehr elderly health (BEH) program

**DOI:** 10.1186/s12889-023-16288-1

**Published:** 2023-08-17

**Authors:** Akram Farhadi, Hamed Javadian, Hakimeh Vahedparast, Maryam Marzban, Reza Nemati, Bagher Larijani, Iraj Nabipour

**Affiliations:** 1 The Persian Gulf Tropical Medicine Research Center, The Persian Gulf Biomedical Sciences Research Institute, Bushehr University of Medical Sciences, Bushehr, Iran; 2 Student Research Committee, Bushehr University of Medical Sciences, Bushehr, Iran; 3 Department of Nursing, School of Nursing and Midwifery, Bushehr University of Medical Sciences, Bushehr, Iran; 4 Clinical Research Development Center, The Persian Gulf Martyrs Hospital, Bushehr University of Medical Sciences, Bushehr, Iran; 5 Department of Human Genetics, McGill University, Montreal, QC Canada; 6 Department of Medical Emergencies, School of Allied Medical Sciences, Bushehr University of Medical Sciences, Bushehr, Iran; 7 Endocrinology and Metabolism Research Center, Endocrinology and Metabolism Clinical Sciences Institute, Tehran University of Medical Sciences, Tehran, Iran; 8 The Persian Gulf Marine Biotechnology Research Center, The Persian Gulf Biomedical Sciences Research Institute, Bushehr University of Medical Sciences, Bushehr, Iran

**Keywords:** Depression, Hypertension, Systolic, Diastolic, Elderly

## Abstract

**Introduction:**

Depression and increased blood pressure are significant burdens in elderly care. This study was conducted to discover the association between hypertension (HTN) and depression based on data obtained from the Bushehr Elderly Health (BEH) program in a large population of Iranian elderly in Bushehr, southern Iran.

**Methods:**

This study was carried out based on data obtained from the Bushehr Elderly Health (BEH) program in a large population of Iranian elderly in Bushehr, a southern city in Iran. 2419 old adults were included in the study through multi-stage random sampling. Depression was assessed using Patient Health Questionnaire-9 (PHQ-9), and blood pressure was measured using a standard mercury sphygmomanometer. Statistical analysis was conducted via chai-square, analysis of variance (ANOVA), and univariate and multivariate linear regression tests.

**Results:**

The mean age of participants was 69.95 ± 6.95 years. The prevalence of depression was 23.8%. Diastolic blood pressure (DBP) decreased with increasing PHQ score (B=-0.001; 95%CI: -0.00 to -0.00, P-value = 0.011). In the final model adjusted for confounding variables, no significant association was found between depression score and DBP (B=-0.00; 95%CI: -0.00 to 0.00, P = 0.13). Conversely, in the final model, which included the confounding variable, SBP was significantly associated with depression. It was deduced that a variable acted as a negative confounder in this association; in a way that with increased depression score, SBP significantly decreased (B=-0.00; 95% CI: -0.00 to -0.00, P = 0.04).

**Conclusion:**

Depression and its related medications could be significantly associated with controlled SBP. Health practitioners in primary health care centers must monitor the elderly inflicted with HTN for early symptoms of depression to help maintain blood pressure levels using medicinal and non-medicinal interventions.

## Introduction

Increased life expectancy does not necessarily equal improvement in life quality. The increased population of elderly around the world has affected various aspects of the quality of life in this population. The elderly face different challenges associated with mental and physical modifications. Numerous studies have postulated direct and indirect associations between physical and psychological health [1]. The incidence of psychological disorders, such as depression, remarkably increases in the elderly [2]. Depression has been introduced as the most prevalent psychological disorder in the elderly and the most prominent disability factor worldwide [3, 4]. Depression also has been suggested as a significant risk factor for disability and mortality in elderly patients [5]. Based on the most recent systematic review, the prevalence of depression in the elderly is around 54% [6]. Despite being common among psychological disorders, depression remains undiagnosed in 50% of the cases [7]. Overall, mental disorders seem more complicated in Asian countries compared to Western societies. In Middle Eastern nations such as Iran, cultural obstacles, including misconceptions, stigmas, discrimination, and poor health literacy, prevent people from seeking help from health professionals [8]; thus, infliction with these diseases could bring negative physical, psychological, and social consequences [9]. The hypothetical impact of psychological factors (such as depression) on blood pressure has been postulated in the existing literature [10]. High blood pressure or hypertension (HTN) is a significant global health challenge, the prevalence of which increases with aging. In fact, more than half of the elderly 60 years or older are affected by this disease [11, 12]. Studies have reported a steady increase in the prevalence of HTN among the elderly over the years [13].

Epidemiologic studies have reported conflicting results concerning the association between depression and HTN [14, 15]. Some studies have shown that blood pressure is higher in people inflicted with depression than in healthy individuals [16]. However, some studies have suggested that depression is associated with lower blood pressure [17–19]. Moreover, some studies have proposed a two-way association between depression and increased blood pressure which eventually will lead to decreased compliance with treatment, lowered quality of life, and augmented mortality in the elderly [14, 20]. The findings from a systematic review and meta-analysis of 27 randomized controlled trials (RCTs) revealed that compared with routine antihypertensive medications, a combined treatment (administration of both antidepressant and antihypertensive agents) results in a significant improvement of systolic and diastolic blood pressure (SBP and DBP).

Based on these studies, the relationship between blood pressure and depression is ambiguous, and most of the studies that investigate this relationship have been conducted in developed countries, while similar studies are insufficient in Middle Eastern countries, such as Iran. In addition, the elderly population is rising due to the increase in life expectancy, which highlights the necessity of planning to improve the quality of life of this population. Therefore, conducting studies with a comprehensive study approach could establish a source of evidence to be used in policymaking and planning in primary health care, as well as in administrating early interventions for managing the prevalence of chronic diseases in the community. The present study used the data derived from Bushehr Elderly Health (BEH) program to investigate the association between depression and HTN in the Iranian elderly population.

## Methods

### Study design and participants

The present cross-sectional study was conducted using information from the second phase of the Bushehr Elderly Health (BEH) program in Bushehr (north of the Persian Gulf), Iran. The BEH program is a prospective cohort study aimed at investigating the prevalence of non-communicable diseases and their associated risk factors. The measurements of phase I baseline were conducted from March 2013 to October 2014. The second phase started in October 2015 and data collection was designed at 2.5-year intervals. Participants in the BEH program were selected using a multi-stage cluster random sampling method. Based on the classifications made by the municipality, we divided the city of Bushehr into 75 districts. A number was assigned to each district, and then the numbers were randomly arranged. We invited all eligible older people living in each selected district to participate in the study and then moved on to the next district and repeated the invitation process to obtain the required sample for that stratum. The sample size for strata was determined according to the number of households living in each stratum. In the second stage, out of 3000 participants having been selected in the first stage, 2419 people were considered eligible to enter the study. Study protocols have been separately published elsewhere [21, 22]. Inclusion criteria were consent, age equal to or above 60 years, residence in Bushehr for at least one year before the start of the study, and not having any plans to leave the city for two years following participation in the study. Also, written and verbal consent was obtained from the participants. The needed information was acquired from medical history, clinical assessment, and in-person interviews. In order to elicit data regarding participants’ medical history, medication, demographic status, physical activity levels, and smoking status, validated questionnaires were used. Moreover, blood samples were obtained to be used in laboratory tests, details of which have been previously published [23]. Trained nurses conducted the medical examination; the related data was registered along with laboratory test results in designated files [23].

### Blood glucose measurements

Infliction with diabetes was verified based on the individual’s self-report, fasting blood sugar (FBS), hemoglobin A1c (HbA1c), and the use of anti-diabetic drugs.

### Blood pressure measurement

Participants were advised to avoid the following activities for at least one hour before blood pressure measurement: vigorous exercise, eating, drinking anything other than water, smoking, taking medications that affect blood pressure. Having a full bladder affects blood pressure and each participant was informed about it. Blood pressure was measured in a non-fasting state. Each person was asked to take out his/her outer coverings such as jacket, coat, etc. and roll up his/her right sleeve, as the blood pressure cuff was to fasten on the right arm. The clothes should not be tight and the cuffs should not be tied on the clothes. If the clothes were tight, they were taken out. The examination was performed in a quiet room with a suitable temperature. The blood pressure cuff was 12.5–12 cm wide and long enough to cover at least two thirds of the arm. If the cuff was short for the person, a larger cuff was used. Blood pressure was measured after the participant had a 5-minute rest without changing the position. The person’s hand was placed on the table in a comfortable position so that the antecubital fossa was at the level of the heart. To get this position, either the seat had to be adjusted or the arm was moved upward or downward. The person remained seated in an upright comfortable position during the examination. The cuff was fastened tightly enough so that it would not slip. Rubber tubes were placed symmetrically on each side of the cubital fossa (the central part of the rubber bag covers the brachial artery). The lower edge of the cuff should be 2–3 cm above the cubital fossa so that there is enough space to insert the diaphragm of the stethoscope. The upper edge of the cuff should not be compressed by clothes. The sphygmomanometer was placed in a suitable position relative to the examination table. The mercury column of the sphygmomanometer should be completely vertical and its center should be at the level of the examiner’s eyes. The person rested in this position for 5 min. At this time, blood pressure measurement procedures were explained to him. If it was necessary for the participant to speak, he/she should keep calm because speaking with great excitement increases the blood pressure [24].

SBP and DBP were measured twice, separated by a fifteen-minute interval using a standard mercury sphygmomanometer (calibrated semi-automatic sphygmomanometer Omron HEM-705CP), on the right arm and in a sitting position. The mean of the two measurements (SBP and DBP) were considered as the participant’s blood pressure.

### Depression

Infliction with depression was assessed using Patient Health Questionnaire-9 (PHQ-9) for all participants. PHQ-9 is a self-reported, nine-item assessment tool that can be used in identifying depression as well as categorizing its severity [25]. This questionnaire has been previously validated in Iran and translated into Farsi [26]. Participants were interviewed regarding the use of antidepressants in the preceding month, and self-reported data was gathered. Individuals were categorized into three levels, including (1) not being depressed (not having any history of depression and not using antidepressants), (2) untreated depression (depression without taking antidepressants), and (3) treated depression (depression along with taking one of the following medications: selective serotonin reuptake inhibitors (SSRIs), non-selective monoamines reuptake inhibitors, monoamine oxidase A inhibitors (MAOIs), antidepressants combined with psychedelics, and other antidepressant medications).

### Covariates

The following covariates were considered in the present study: age (years); gender (male/female); marital status (single, married, divorced, widow); body mass index (BMI) (weight (kg)/ [height (m)^2^]); education (primary school, high school, diploma, academic); diabetes (defined as self-reported physician diagnosis, medication use, fasting blood glucose ≥ 126 mg/dL, or non-fasting blood glucose ≥ 200 mg/dL); dyslipidemia; smoking status (current or past use of cigarettes or hookah); physical activity (sedentary, moderately active, active, very active, and extremely active); the use of anti-hypertension drugs, including angiotensin-converting enzyme inhibitors (ACEIs), angiotensin receptor blockers (ARBs), alpha-blocker medications, beta-blockers, calcium channel blockers (CCBs), diuretic medications, and nitrates medications; cognitive impairment; use of anti-depression drugs; and use of anti-hyperlipidemia drugs.

Essential variables were selected based on univariate statistical significance and literature review. They were then entered into the models based on their presumed effectiveness. The following models were fitted: Model 1: age, education, and BMI; Model 2: Model 1 + diabetes and dyslipidemia; Model 3: Model 2 + antihypertensives, antidepressants, and lipid-lowering agents; Model 4: Model 3 + physical activity, smoking cigarettes, and cognitive disorders; and Model 5: Model 4 + biological gender.

### Statistical analysis

String variables were presented as frequency and percentage, while nominal variables were demonstrated as mean ± standard deviations (SDs). Bivariate analyses included chi-square and analysis of variance (ANOVA) tests. Univariate and multivariate regressions were used to estimate the odds ratios (ORs) between depression and blood pressure management. Based on the literature, five models were fitted in multivariate analyses. Statistical analyses were conducted using STATAMP 15 software. Statistical significance was approved with p-values < 0.05. 95% confidence intervals (CIs) were reported.

### Ethical considerations

BEH program protocol was approved by the research ethics committees of Bushehr and Tehran medical universities under the registration code (IR.TUMS.EMRI.REC.1394.0036). Moreover, the ethics code (IR.BPUMS.REC.1399.055) was acquired from the regional bioethics committee of Bushehr medical university. It was explained to the eligible individuals that participation was voluntary and, based on privacy principles, their first and last names were not registered in the questionnaires. Informed consent was obtained based on the declaration of Helsinki for medical research.

## Results

The average age of participants was 69.95 ± 6.95 years. The prevalence of depression was 23.8%. Even though most people with depression did not use antidepressants or other therapies, 50 participants suffering from depression used antidepressant agents. Interestingly, the percentage of female individuals, either taking or not taking antidepressant agents was significantly more than male individuals (p-value < 0.001). Moreover, in people with untreated depression, cigarette and hookah smoking was higher than in other groups. Even though the physical activity level (PAL) was assessed to be very low and even sedentary, it was significantly lower in the Not Treated Depression (NTD) group compared to the two other groups. A significant percentage of individuals used antihypertensive agents but treated HTN was more frequent in the Treated Depression (TD) group. However, the portion of individuals with treated HTN was lower in the NTD group (Table 1).

**Table 1:** Main characteristics of study population by depression status: Bushehr Elderly Health (BEH) program (n = 2419)

Characteristics of participants		No Depression (1784)	Depression with Treatment (50)	Depression without Treatment (573)	P-value
Mean age (years)*		69.19 ± 6.21	67.62 ± 5.83	69.98 ± 6.95	0.007*
Mean BMI (kg/m2) *		27.32 ± 4.70	28.51 ± 5.30	27.97 ± 5.42	0.0050*
sex (%)	Males	1012 (56.73)	11 (22.00)	138 (24.08)	0.000**
	Females	772 (43.27)	39 (78.00)	435 (75.92)	
Marital status, n (%)	Single	10 (0.56)	-	7 (1.12)	0.000**
	Married	1,439 (80.66)	34 (68.00)	415 (66.61)	
	Divorce	10 (0.56)	-	10 (1.61)	
	Widow	325 (18.22)	16 (32.00)	191 (30.66)	
Educational attainment	Primary School	667 (37.39)	19 (38.00)	193 (33.68)	0.000**
	High school	172 (9.64)	9 (18.00)	37 (6.46)	
	Diploma	276 (15.47)	6 (12.00)	50 (8.73)	
	Academic	167 (9.36)	-	19 (3.32)	
Smoking	None	561 (31.45)	22 (44.00)	149(26.00)	0.004**
	Past cigarette or hookah	875 (49.05)	19 (38.00)	280 (48.87)	
	current cigarette or hookah	348 (19.51)	9 (18.00)	144 (25.13)	
Physical activity, n (%)	Sedentary	1240 (73.77)	35 (70.00)	417 (79.28)	0.000**
	Low active	264 (15.70)	6(12.00)	79 (15.02)	
	Active	84 (5.00)	1(2.00)	22 (4.18)	
	Very active	33 (1.94)	-	4 (0.76)	
	More Than	60 (3.57)	-	4 (0.76)	
Classification of BP	Normotensive	93(5.23)	3(5.88)	46(7.38)	0.027
	Prehypertensive	364(20.46)	10(19.61)	94(15.09)	
	Hypertensive	1322(74.31)	38(74.51)	483(77.53)	
SBP (mmHg)	SBP1	140.69 ± 20.07	135.14 ± 20.59	140.41 ± 21.52	0.141
	SBP2	139.09 ± 19.33	132.88 ± 20.58	137.80 ± 20.24	0.056
	Mean SBP	139.89 ± 19.06	134.01 ± 20.03	139.55 ± 20.55	0.090
DBP (mmHg)	DBP1	81.70 ± 9.40	81.44 ± 10.86	81.13 ± 9.83	0.422
	DBP2	81.86 ± 9.05	80.54 ± 9.54	80.77 ± 9.41	0.030
	Mean DBP	81.78 ± 8.64	80.99 ± 9.50	80.95 ± 8.68	0.112
History of HTN	Yes	732(51.99)	17(54.84)	276(61.74)	0.001
	No	676(48.01)	14(45.16)	171(38.26)	
Antihypertensive treatment	Yes	882(61.59)	31(62.00)	321(64.46)	< 0.001
	No	550(38.41)	19(38.00)	177(35.54)	
Treated HTN	Yes	1181(66.39)	34(68.00)	374(60.03)	0.012
	No	598(33.61)	16(32.00)	249(33.97)	
Cognitive Impairment	Yes	802(44.96)	33(66.00)	349(60.91)	< 0.001
	No	982(55.04)	17(34.00)	224(39.09)	
Sedative	Yes	103(7.19)	24(48.00)	67(13.45)	< 0.001
	No	1329(92.81)	26(52.00)	431(86.55)	
Dyslipidemia	Yes	1213(67.77)	34(66.67)	410(70.69)	0.325
	No	577(32.23)	17(33.33)	170(29.31)	
Diabetes	Yes	904(50.67)	31(62.00)	334(58.49)	0.001
	No	880(49.33)	19(38.00)	237(41.51)	

All values are reported as mean ± (SE or SD) were derived from ANOVA

*ANOVA,

**chi-square

The significance level was considered P < 0.05.

SBP was found to be lower in the TD group compared to the non-depressed (ND) group (B=-0.49; 95%CI: -0.87 to -0.011, P = 0.011) (Table 2). Moreover, treatment with sedatives reduces SBP and DBP (B=-0.03, p-value: 0.002; and B=-0.018, p-value = 0.027, respectively). We also found that infliction with diabetes mellitus was associated with increased SBP (B = 0.19; 95% CI: 0.007 to 0.030, P = 0.002). On the other hand, we showed that the use of antidepressants was associated with decreased SBP (P = 0.001); however, we failed to detect such an association with DBP (P = 0.811). Furthermore, people with cognitive disorders had a higher propensity to have increased SBP (B = 0.012; 95% CI: 0.00 to 0.024, P = 0.041), while we could not show a similar association with DBP (B=-0.002, 95% CI: -0.11 to 0.007, P = 0.635) (Table 2).

**Table 2:** Log-linear model coefficients of systolic and diastolic blood pressure according to depression level and adjusted co-variables (n = 2419)

			Natural logarithm of systolic blood pressure(mmHg)				Natural logarithm of diastolic blood pressure (mmHg)					
Characteristics of participants			**Coefficients**	**Lower CI 95%**	**Upper CI 95%**	**p-value**	**Coefficients**	**Lower CI 95%**		**Upper CI 95%**		**p-value**
Depression level		No Depression	Ref	Ref	Ref	Ref	Ref	Ref		Ref		Ref
		depression with medication	-0.049	-0.087	-0.011	0.011	-0.010		-0.041		0.020	0.509
		Depression without medication	-0.004	-0.018	0.009	0.542	-0.009	-0.020		0.001		0.104
Anti-Anxiety or sedative treatment			-0.030	-0.050	-0.010	0.002	-0.018	-0.034		-0.002		0.027
Age(years)			0.000	0.000	0.000	0.032	-0.000	-0.000		0.000		0.000
Sex (female)			0.003	-0.008	0.015	0.597	0.019	0.009		0.028		0.000
Educational attainment		Primary School	-0.013	-0.027	0.000	0.067	0.004	-0.006		0.016		0.419
		High school	-0.008	-0.030	0.013	0.447	0.028	0.010		0.046		0.002
		Diploma	-0.027	-0.045	-0.008	0.005	0.002	-0.013		0.017		0.775
		Academic	-0.045	-0.069	-0.021	0.000	0.001	-0.018		0.020		0.893
Body Mass Index(kg/m2)			0.003	0.002	0.005	0.000	0.003	0.002		0.004		0.000
Smoking		Cigarette	-0.010	-0.020	-0.000	0.040	-0.001	-0.009		0.006		0.759
		Hookah	-0.005	-0.014	0.002	0.185	-0.002	-0.009		0.003		0.394
		Cigarette or hookah	-0.006	-0.015	0.001	0.112	-0.003	-0.009		0.003		0.374
Diabetes			0.019	0.007	0.030	0.002	-0.008	-0.017		0.001		0.102
Dyslipidemia			0.001	-0.009	0.013	0.765	0.001	-0.007		0.010		0.738
Antidepressant			-0.044	-0.069	-0.018	0.001	-0.002	-0.023		0.018		0.811
Antihyperlipidemic drug			-0.005	-0.017	0.006	0.408	-0.009	-0.019		0.000		0.056
Physical activity	Sedentary		-0.017	-0.041	0.006	0.149	0.003	-0.016		0.022		0.743
	Low Active		-0.027	-0.055	-0.000	0.045	-0.003	-0.025		0.018		0.762
	Active		-0.017	-0.052	0.016	0.313	0.004	-0.022		0.032		0.729
	Very Active		-0.043	-0.102	0.014	0.141	-0.018	-0.065		0.028		0.439
Cognitive Impairment			0.012	0.000	0.024	0.041	-0.002	-0.011		0.007		0.635

All values are reported as Coefficients and 95% confidence interval were derived from Log-linear model.

The significance level was considered P < 0.05.

The adjusted model for potential confounders is presented in Table 3. This model shows that when the model is fitted with depressions score as a continuous variable, an increased PHQ-9 score is associated with reduced DBP (B=-0.001; 95% CI: -0.00 to 0.00, P = 0.011). However, it should be noted that even though the association seems to be statistically significant, the correlation coefficient (β) is negligible. Due to this fact, in the final model in which other covariates (i.e., age; education; BMI; infliction with diabetes mellitus, dyslipidemia, and cognitive disorders; use of antihypertensive, antidepressant, and lipid-lowering agents; physical activity; and biological sex) were entered, the association between depression score and DBP vanished (B=-0.00; 95%CI: -0.00 to 0.00, P = 0.133). This observation suggests there exists a potent enough confounder capable of nullifying this association. Conversely, in the crude model, the depression score was not associated with SBP (B=-0.00; 95% CI: -0.01 to 0.00, P = 0.103). However, when potential confounders were entered into the model, the latter association turned significant (B=-0.00; 95% CI: -0.00 to -0.00, P = 0.047). Nonetheless, the observed associations are minimal (Table 3).

**Table 3:** The relationship between depression & systolic and diastolic blood pressure; Bushehr Elderly Health (BEH) program (n = 2419)

	Diastolic BP				Systolic BP				
Models	**Coefficients**	**Lower CI 95%**	**Upper CI 95%**	**p-value**		**Coefficients**	**Lower CI 95%**	**Upper CI 95%**	**p-value**
Unadjusted	-0.001	-0.002	-0.0002	0.011		-0.005	-0.011	0.0010	0.103
Model1	-0.001	-0.002	-0.0004	0.005		-0.001	-0.003	-0.0007	0.001
Model2	-0.001	-0.002	-0.0004	0.005		-0.002	-0.003	-0.0008	0.001
Model3	-0.001	-0.002	-0.0000	0.040		-0.001	-0.002	-0.0002	0.019
Model4	-0.001	-0.002	-0.0004	0.009		-0.002	-0.003	-0.0005	0.007
Model5	-0.0009	-0.002	0.0002	0.133		-0.001	-0.003	-0.0000	0.047

All values are reported as Coefficients and 95% confidence interval were derived from Multiple linear regression.

Model 1: adjusted for age, education, and BMI.

Model 2: adjusted for Model 1 + diabetes, dyslipidemia

Model 3: adjusted for Model 2 + anti hypertension drugs, anti-depression drugs, and antihyperlipidemic drugs.

Model 4: adjusted for Model 3 + physical activity, smoking status, cognitive impairment.

Model 5: adjusted for Model 4 + sex

The significance level was considered P < 0.05.

## Discussion

The present study aimed to investigate the association between depression and HTN in the elderly participating in the BEH program. Our findings suggest that even though depression score was associated with SBP after controlling for the effect of possible confounders, no clinically relevant association exists between these two variables. We believe that there is a covariate that plays a decisive confounding role in the association between depression and blood pressure. Despite controlling for potential confounders in the multivariate analysis, the association between depression and blood pressure control is independent. However, the findings of similar studies have also suggested the ambiguity of such an association [10]. In the present study, confounders possibly associated with depression and HTN were considered in the analysis, including age; education; BMI; infliction with diabetes mellitus, dyslipidemia, and cognitive disorders; physical activity; and biological sex. Some mechanisms may be enumerated to explain the facilitated blood pressure management in individuals with depression, such as suppressed vagal activity, reduced heart rate, baroreflex sensitivity, and modified neuro-endocrine pathways caused by the use of antidepressant agents [27–30]. Nevertheless, some studies have reported opposite findings, suggesting that depression might be an independent risk factor in the pathophysiology of HTN [31, 32].

We also found that although many older people were suffering from depression, they still did not receive any treatment. Many reasons have been reported for avoiding antidepressants, such as fear of their side effects, taking too many drugs, or medication addiction. Also, many deny their condition and believe that depression does not exist or that there is no need for drug treatment. The fear of the stigma of being labeled or embarrassed also prevents them from seeking further care and pushes them toward the wait-and-see approach [33].

In the present study, we found that the prevalence of depression was higher in women than in men. This observation might be justified by hormonal fluctuations, the conception of stress, sexual discrimination, and socio-economic hurdles women are more likely to face. Additionally, women have been shown to be far more vulnerable to physical and psychological injuries caused by daily-life stress [6, 34]. Other studies have reported similar findings for the different prevalence’s of depression in men and women [6, 35].

This study also showed that cigarette and hookah use was more common in the elderly with untreated depression. Some mechanisms may be responsible for this regard; for instance, it has been postulated that with advanced age, the central nervous system might lose its capacity to maintain its function despite hormonal fluctuations. As a result, the elderly who experience more anxiety are more prone to produce higher levels of stress hormones which might not rapidly plunge [36]. Stress is the leading cause of smoking, and people who are stressed are more prone to smoking. Smoking has been said to help smokers cope with a negative affective state, and they often report that smoking reduces their tension and relaxes them [37, 38]. Moreover, individuals with more suited coping mechanisms are less likely to be affected by these adverse conditions [39]. Smoking is an unhealthy coping strategy that merely reduces the symptoms of stress without the sources of the stress being addressed [40]. Studies have clearly shown that smoking increases the risk of depression and does not play a positive role in combating negative mood [41, 42].

Our study also suggests that the elderly with untreated depression are less physically active. This finding was well anticipated, given that the antidepressant agents are believed to improve mood, joy, and level of physical activity [43]. On the other hand, the results of other studies have also suggested that the lack of physical activity is associated with an increased risk of psychological disorders, including anxiety and depression [44, 45].

Previous studies have proposed that underlying conditions, such as diabetes mellitus, are related to increased SBP. This association has been justified by the vascular damage caused by diabetes mellitus, leading to atherosclerosis and increased blood pressure [46]. Genetics, obesity, psychological stress, sympathetic stimulation, and insulin resistance are among the etiologies hypothesized for increased blood pressure in people with diabetes mellitus [47]. Also, the destruction of kidney nephrons, one of the major microvascular complications of diabetes mellitus, causes increased fluid volume in the body, eventually leading to increased blood pressure [48].

We also signified cognitive disorders in predicting the increased SBP. These results are echoed by the findings of Forte et al. (2019), and Reitz et al. (2007) reported that increased blood pressure was associated with a higher risk of cognitive disorders [49, 50].

The present study’s findings also showed that even though a higher percentage of individuals in the NTD group received HTN medications, treated HTN was more prevalent in the TD group. Given that the TD group individuals were more likely to receive sedative agents, the preceding association might be caused by the blood pressure-lowering impact of anti-anxiety and sedative agents. Moreover, it has been shown that the use of antidepressants also causes a reduction in SBP in the elderly while not significantly impacting DBP. Our findings align with previous studies that reported that the consumption of antidepressant and sedative agents, separately or combined, can reduce blood pressure [51, 52]. It could also be deduced that frequent use of health care services in the TD group might have helped improve their blood pressure and other comorbidities. Multivariate analysis showed that the association between depression and blood pressure is independent, as previously stated in several studies [53, 54]. However, few studies have proposed that blood pressure was lower in the elderly with HTN who received antihypertensive agents [55]. Demirtürk et al. [35] have shown that the early diagnosis of and intervention against depression is a substantial factor in managing HTN.

The present study had some limitations. Firstly, the study’s cross-sectional design renders deriving a cause-and-effect relationship impossible. Secondly, the two-time blood pressure measurement might not accurately represent chronic infliction with HTN. We propose that further longitudinal studies, considering all potential confounders, be conducted to unravel the mechanisms explaining the association between depression and increased blood pressure.

## Conclusion

The present study’s findings indicate that depression and its related therapeutics are associated with improved SBP. Accordingly, it seems crucial that family physicians put more effort into a psychological screening of the elderly for early diagnosis and intervention of depression. In Iran and similar countries with analogous cultural backgrounds, symptoms of depression are regarded as the “natural” process of aging; therefore, its diagnosis and treatment are largely undermined. Thus, it is required that more attention be paid to this prospect of health in the elderly. Hence, enhancing the awareness of the elderly and their families concerning mental health and increasing the availability of community-based mental health programs could help improve the timely diagnosis and intervention of depression and its presumed comorbidities, such as HTN. Additionally, it is crucial for health practitioners in primary health care centers to screen the elderly with chronic diseases, including HTN, for signs and symptoms of depression to facilitate the early diagnosis and suited medicinal and non-medicinal therapies for depression. Which would, in turn, help manage and ameliorate HTN in this section of society.

## Data Availability

The datasets used and/or analyzed during the current study are available from the corresponding author on request.
